# Comparative Histopathological Effects of Intra-Articular Bevacizumab, Ranibizumab, and Aflibercept in Experimental Osteoarthritis

**DOI:** 10.3390/jcm15176565

**Published:** 2026-08-25

**Authors:** Raşit Emin Dalaslan, Mehmet Arıcan, Zekeriya Okan Karaduman, Mücahid Osman Yücel, Sönmez Sağlam, Fatih Demir, Mücahit Çelik

**Affiliations:** 1 Orthopedics and Traumatology Department, Duzce University Medical Faculty, Duzce 81620, Turkey; ari_can_mehmet@hotmail.com (M.A.); karadumano@hotmail.com (Z.O.K.); mucahidyucel@duzce.edu.tr (M.O.Y.); dr.sonmezsaglam@gmail.com (S.S.); 2 Department of Pathology, School of Medicine, Duzce University, Duzce 81620, Turkey; fatihfnd@gmail.com; 3 Department of Orthopaedics and Traumatology, Balıkesir Atatürk City Hospital, Balıkesir 10100, Turkey; mucahitcelik1994@hotmail.com

**Keywords:** osteoarthritis, angiogenesis, vascular endothelial growth factor, bevacizumab, ranibizumab, aflibercept, experimental osteoarthritis, animal model

## Abstract

**Background**: Vascular endothelial growth factor (VEGF) has emerged as a potential therapeutic target in osteoarthritis (OA). Although several anti-VEGF agents are widely used in clinical practice, their comparative effects on osteoarthritic cartilage remain unclear. This study compared the histopathological effects of intra-articular bevacizumab, ranibizumab, and aflibercept in an experimental rat model of OA. **Methods**: Experimental OA was induced in 36 rats using anterior cruciate ligament transection. One month later, animals received two intra-articular injections of bevacizumab (2 mg/kg), ranibizumab (0.5 mg/kg), aflibercept (3.2 mg/kg), or saline at 3-week intervals. Histopathological evaluation was performed using the Osteoarthritis Research Society International (OARSI) grading and staging system by two blinded pathologists. The primary outcome was the total OARSI score. Group comparisons were performed using the Kruskal–Wallis test followed by Dunn–Holm post hoc analysis. **Results**: Three animals were lost during anesthesia and surgical induction of OA before treatment allocation, leaving 33 rats for analysis. Significant overall differences were observed among the groups for the primary outcome of total OARSI score (*p* = 0.026) and the secondary outcomes of OARSI grade (*p* = 0.042) and OARSI stage (*p* = 0.019). Ranibizumab demonstrated numerically lower OARSI grade and total scores than the control group, whereas both groups had the same median OARSI stage score. Bevacizumab showed numerically lower OARSI grade and total scores than the control group but a slightly higher median stage score. Pairwise analysis showed significantly lower OARSI stage and total OARSI scores in the ranibizumab group than in the aflibercept group, whereas no significant differences were observed between any treatment group and the control group after correction for multiple comparisons. **Conclusions**: Anti-VEGF agents demonstrated differential histopathological responses in experimental OA. Although no treatment significantly improved histopathological outcomes compared with the control group after adjustment for multiple comparisons, ranibizumab showed lower OARSI stage and total scores than aflibercept. These findings indicate that the evaluated anti-VEGF agents may not exert equivalent biological effects and warrant further investigation in adequately powered experimental studies.

## 1. Introduction

Osteoarthritis (OA) is the most common chronic musculoskeletal disorder and a leading cause of pain, disability, and reduced quality of life worldwide [1,2]. Rather than being a disease confined to progressive articular cartilage degeneration, OA is now recognized as a complex whole-joint disorder driven by interacting mechanical, inflammatory, metabolic, and cellular processes [3,4]. Pathological changes develop not only in the articular cartilage but also in the subchondral bone, synovium, menisci, ligaments, periarticular muscles, and infrapatellar fat pad, resulting in progressive structural deterioration and loss of joint function [3,4,5]. As population aging and obesity continue to increase globally, the socioeconomic burden associated with OA is expected to rise substantially, highlighting the need for more effective disease-modifying therapeutic strategies [1,2].

Increasing evidence indicates that abnormal biomechanical loading and impaired mechanotransduction play central roles in OA initiation and progression [4,6]. Mechanical stress disrupts extracellular matrix homeostasis, promotes chondrocyte phenotypic changes, and stimulates the production of inflammatory cytokines, matrix-degrading enzymes, and angiogenic mediators [3,4,7]. These events contribute to reciprocal interactions among cartilage, synovium, subchondral bone, menisci, ligaments, and the infrapatellar fat pad, which is increasingly recognized as an active immunometabolic organ capable of secreting adipokines, inflammatory mediators, and pro-angiogenic factors that further amplify joint degeneration [4,5]. This contemporary understanding supports the concept that OA is a disease of the entire joint microenvironment rather than isolated cartilage degeneration [3,4]. Among the mediators implicated in these processes, vascular endothelial growth factor (VEGF) has emerged as a critical regulator of OA progression [3,8].

Normal adult articular cartilage is an avascular tissue; however, VEGF expression is markedly increased in osteoarthritic cartilage, synovium, synovial fluid, and subchondral bone. Increased VEGF expression has been associated with cartilage matrix degradation, osteophyte formation, synovial inflammation, angiogenesis, pain severity, and radiographic disease progression. These observations suggest that VEGF signaling plays an important role in OA pathogenesis and may represent a promising therapeutic target [7,9,10].

Bevacizumab, ranibizumab, and aflibercept are clinically established anti-VEGF agents widely used in the management of angiogenesis-related diseases [11,12]. Bevacizumab has been extensively used in both oncology and ophthalmology, whereas ranibizumab is approved exclusively for intravitreal treatment of retinal vascular diseases [11,13]. Aflibercept is used in ophthalmology and has also been developed for selected oncological indications [12,13]. Although all three agents inhibit VEGF signaling, they differ in molecular structure, VEGF-binding characteristics, pharmacokinetic profiles, and biological activity, factors that may influence their therapeutic effects in musculoskeletal tissues [11,13].

Several experimental studies have demonstrated that VEGF inhibition may attenuate synovial angiogenesis, reduce cartilage degeneration, and preserve joint architecture in OA models [10]. In particular, intra-articular bevacizumab has been reported to improve histopathological findings and suppress structural progression of experimental OA, supporting the concept that VEGF inhibition may represent a potential disease-modifying strategy for OA [14].

Nevertheless, whether different anti-VEGF agents provide comparable protective effects on osteoarthritic cartilage remains unclear. To date, direct head-to-head comparisons of bevacizumab, ranibizumab, and aflibercept in the same experimental OA model are extremely limited. Therefore, the present study aimed to compare the histopathological effects of intra-articular bevacizumab, ranibizumab, and aflibercept in a surgically induced rat OA model. By directly comparing these three clinically available anti-VEGF agents, this exploratory study sought to determine whether different anti-VEGF agents produce comparable histopathological responses following intra-articular administration and to generate preliminary evidence that may help guide future candidate selection and experimental dosing strategies for OA research.

## 2. Materials and Methods

### 2.1. Study Design

This experimental animal study was designed to evaluate and compare the histopathological effects of intra-articular bevacizumab, ranibizumab, and aflibercept in a surgically induced rat OA model. All experimental procedures were conducted in accordance with institutional guidelines for the care and use of laboratory animals and were approved by the Düzce University Animal Experiments Local Ethics Committee (meeting date: 12 June 2024; approval no. 2024-06-02). The study was carried out at the Düzce University Experimental Animals Application and Research Center. All procedures were performed in accordance with the ARRIVE 2.0 guidelines for reporting animal research.

### 2.2. Animals

Thirty-six male Wistar Albino rats aged 2–3 months and weighing 230 ± 30 g were included in the study. Male Wistar Albino rats were selected to maintain a relatively homogeneous experimental cohort and reduce sex- and strain-related biological variability within this exploratory comparative study. The animals were obtained from the Düzce University Experimental Animal Research and Application Center. Animals were housed in standard polypropylene cages under controlled environmental conditions (23–25 °C, relative humidity 60 ± 5%, and a 12-h light/dark cycle). Standard laboratory chow and water were provided ad libitum throughout the study. Following a one-week acclimatization period, all animals underwent surgical intervention.

Animals completing the surgical induction of OA were allocated to the four experimental groups using a simple randomization method. Animals were monitored daily throughout the study for general health and welfare, and no predefined humane endpoint requiring euthanasia was reached during the experimental period.

### 2.3. Experimental Osteoarthritis Model

**Figure 1:**
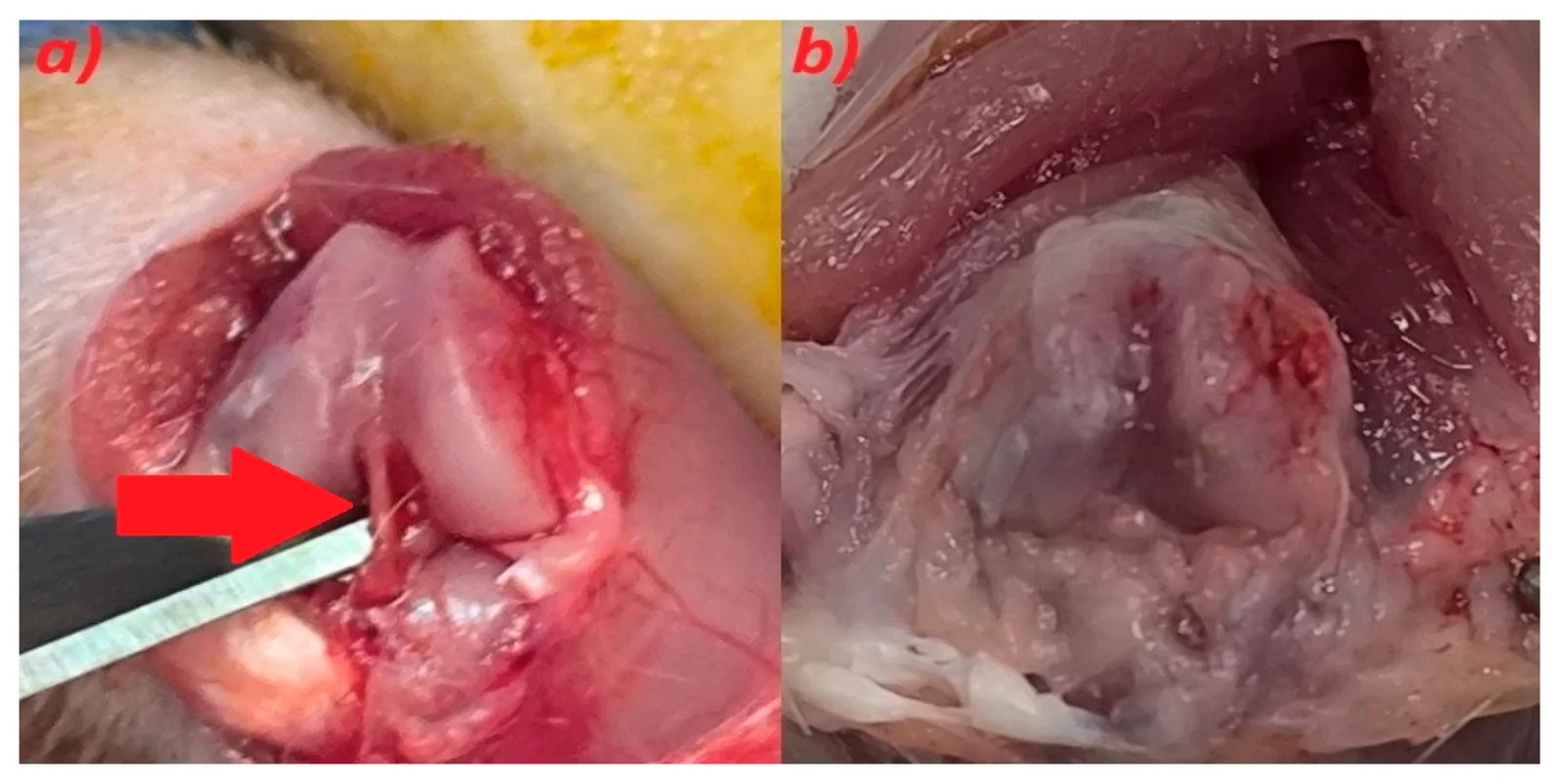
Macroscopic images of the experimental osteoarthritis (OA) model. (**a**) Surgical induction of OA using the anterior cruciate ligament transection (ACLT) technique. The arrow indicates the anterior cruciate ligament (ACL), which is identified under direct visualization before transection. (**b**) Representative macroscopic appearance of the knee joint following experimental OA induction, demonstrating extensive cartilage loss over the femoral condyles, irregular and roughened articular surfaces, and focal areas of exposed subchondral bone, consistent with advanced osteoarthritic degeneration.

Experimental OA was induced using the anterior cruciate ligament transection (ACLT) model [15,16]. Anesthesia was achieved with intraperitoneal ketamine hydrochloride (90 mg/kg) and xylazine (10 mg/kg) [17]. Following sterile preparation of the right knee, a medial parapatellar incision was performed and the patella was laterally displaced to expose the joint cavity. The anterior cruciate ligament was transected under direct visualization, and complete transection was confirmed using the anterior drawer test (Figure 1). The joint cavity was irrigated with sterile saline solution, and the incision was closed anatomically [15]. No postoperative analgesic agents were administered because of concerns that analgesics could influence the inflammatory response and potentially interfere with histopathological evaluation. The study protocol, including the decision to withhold postoperative analgesia, was specifically reviewed and approved by the Düzce University Animal Experiments Local Ethics Committee. Animals were monitored at least twice daily for signs of pain, distress, or impaired mobility throughout the experimental period.

### 2.4. Experimental Groups and Intra-Articular Injections

One month after OA induction, intra-articular injections were administered under sterile conditions. The anti-VEGF agents used in this study were bevacizumab (Bevax^®^, 100 mg/4 mL concentrate for solution for intravenous infusion; Abdi İbrahim Pharmaceuticals, Istanbul, Türkiye), ranibizumab (Lucentis^®^, 10 mg/mL solution for injection; Novartis Pharma AG, Basel, Switzerland), and aflibercept (Eylea^®^, 114.3 mg/mL intravitreal solution for injection; Bayer Türk Kimya San. Ltd., Şti., Istanbul, Türkiye). All medications were commercially available sterile formulations and were administered by intra-articular injection under aseptic conditions.

The bevacizumab group (*n* = 8) received 2 mg/kg bevacizumab in a volume of 0.04 mL.

The ranibizumab group (*n* = 8) received 0.5 mg/kg ranibizumab in a volume of 0.04 mL.

The aflibercept group (*n* = 9) received 3.2 mg/kg aflibercept in a volume of 0.04 mL.

The control group (*n* = 8) received 0.04 mL saline solution.

For each injection, the required drug dose was calculated individually according to the body weight of each animal. The corresponding volume of the commercial formulation was withdrawn based on its stated concentration and diluted with sterile 0.9% saline to a fixed final injection volume of 0.04 mL. Thus, although the final injection volume was identical for all animals, the volume of the commercial drug solution and the amount of added saline varied according to the individual body weight and assigned dose.

The assigned treatments were administered as two intra-articular injections at three-week intervals. Injections were performed under sterile conditions using a 30-gauge needle inserted through the infrapatellar region with the knee in slight flexion. Each injection was administered slowly in a total volume of 0.04 mL. Correct intra-articular placement was confirmed by the absence of resistance during injection and the absence of leakage or visible subcutaneous swelling at the injection site.

The bevacizumab dose (2 mg/kg) was selected based on previously published experimental studies evaluating intra-articular bevacizumab administration in OA models [18]. Because validated intra-articular dosing regimens for ranibizumab and aflibercept are currently unavailable, their doses were determined by preserving the relative dose proportions established among bevacizumab, ranibizumab, and aflibercept in routine intravitreal clinical practice [19]. Accordingly, the ranibizumab (0.5 mg/kg) and aflibercept (3.2 mg/kg) doses were extrapolated from the established bevacizumab dose while maintaining the relative dosing relationship between the three anti-VEGF agents. This approach was adopted to enable a standardized comparative evaluation of their histopathological effects, although direct pharmacokinetic equivalence between intravitreal and intra-articular administration cannot be assumed. Because no established intra-articular doses were available for ranibizumab or aflibercept in experimental OA, the present study was designed as an exploratory single-dose comparison rather than an equimolar or dose–response investigation.

### 2.5. Tissue Sampling and Histological Preparation

At the end of the third postoperative month, all animals were placed under deep anesthesia, and euthanasia was performed by exsanguination via cardiac blood collection. Knee joint specimens including the distal femur, proximal tibia, articular cartilage surfaces, and subchondral bone were carefully dissected while preserving joint integrity.

The specimens were fixed in 10% neutral buffered formalin for 72 h. After fixation, decalcification was performed using 10% formic acid solution. Following confirmation of complete decalcification, tissue samples were dehydrated through graded alcohol series, cleared in xylene, and embedded in paraffin blocks in the coronal plane to optimally demonstrate the weight-bearing articular surfaces.

**Figure 2:**
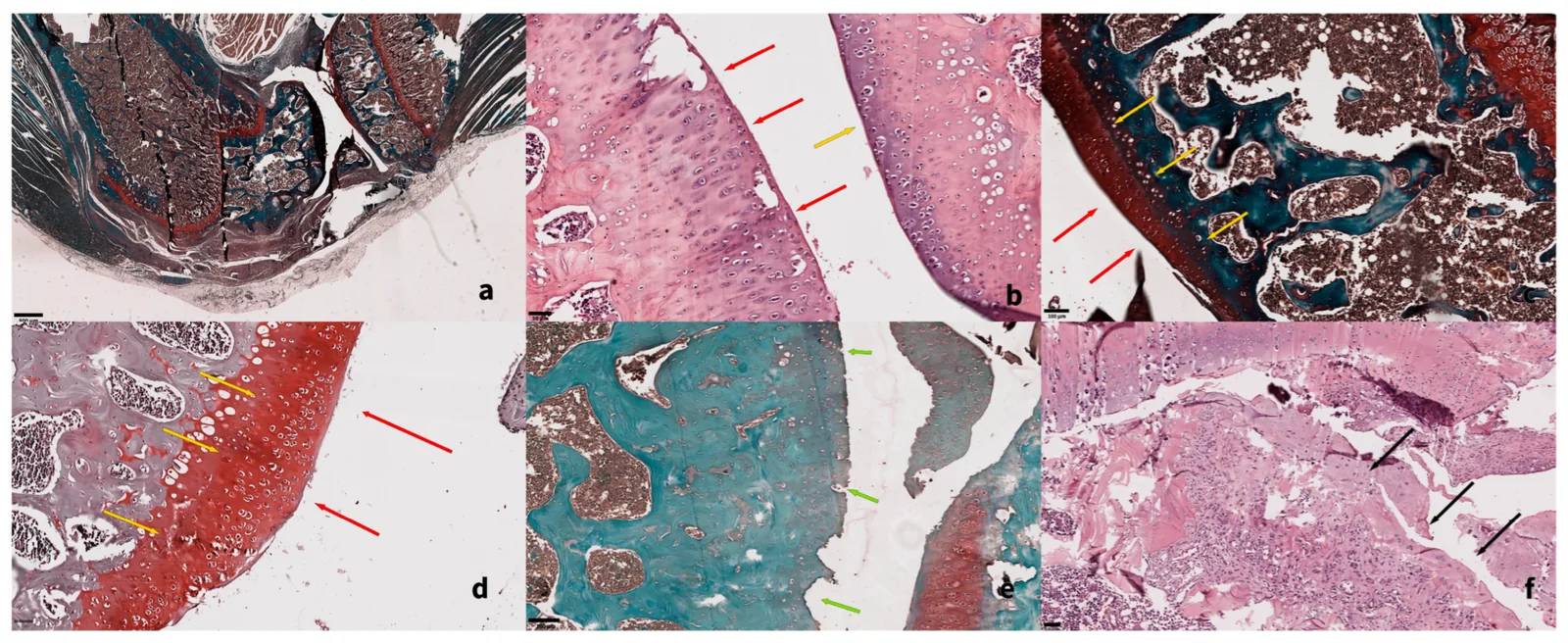
Representative histological sections illustrating osteoarthritic degeneration according to the OARSI histopathological grading and staging system. (**a**) Representative section from the bevacizumab group, OARSI Grade 1 and Stage 2, stained with Safranin O–Fast Green at low magnification, showing preservation of the articular cartilage contour and intense matrix staining. (**b**) Representative section from the ranibizumab group, OARSI Grade 1 and Stage 2, stained with hematoxylin and eosin (H&E) at high magnification, demonstrating preserved cartilage matrix (yellow arrow) and an intact cartilage surface without evident fissures or erosions (red arrows). (**c**) Representative section from the control group, OARSI Grade 1 and Stage 2, stained with Safranin O–Fast Green at high magnification, demonstrating preserved Safranin O staining consistent with retained proteoglycan content (yellow arrows) and an intact articular surface (red arrows). (**d**) Representative section from the aflibercept group, OARSI Grade 2 and Stage 2, stained with Safranin O–Fast Green, showing surface irregularities (red arrows) with mildly reduced but preserved Safranin O staining (yellow arrows). (**e**) Representative section from the ranibizumab group, OARSI Grade 3 and Stage 2, stained with Safranin O–Fast Green, demonstrating a deep vertical cartilage fissure extending into the cartilage matrix (green arrows). (**f**) Representative section from the control group, OARSI Grade 4 and Stage 3, stained with H&E, demonstrating advanced structural disruption with extensive cartilage erosion and defect formation (black arrows) and reparative tissue formation at the base of the defect. Scale bars are shown within each panel.

Serial sections of 5–6 μm thickness were obtained using a microtome. Hematoxylin and eosin (H&E) staining was performed for general morphological evaluation, while Safranin O–Fast Green staining was used to assess cartilage proteoglycan content and matrix degeneration associated with OA. Representative histological sections demonstrating progressive osteoarthritic changes were documented using both staining methods (Figure 2).

### 2.6. Histopathological Evaluation

Histopathological evaluation was independently performed under light microscopy by two blinded pathologists who were unaware of the treatment groups. From each knee joint, a single representative coronal section obtained from the femoral condyle, encompassing the weight-bearing articular cartilage, was selected for histopathological assessment. Because one representative section was evaluated for each specimen, no averaging across multiple tissue sections or selection of a maximum score was performed.

Cartilage degeneration was assessed according to the OARSI histopathological grading and staging system described by Pritzker et al. [20]. A separate rodent-specific OARSI histopathological assessment system has also been developed for rat OA models [21]; however, the Pritzker grade–stage system was used in the present study. OARSI grading (0–6) was based on the severity of cartilage structural damage, ranging from intact cartilage to severe deformation with subchondral bone involvement, whereas OARSI staging (0–4) reflected the horizontal extent of cartilage involvement (<10%, 10–25%, 25–50%, and >50% of the articular surface). The total OARSI score was calculated by multiplying the grade by the stage (Grade × Stage), providing a semiquantitative measure of OA severity [16,20,22].

The primary histopathological outcome was the total OARSI score, whereas OARSI grade and OARSI stage were predefined secondary outcomes. Each pathologist independently assigned OARSI grade and stage scores. When the scores differed between the two observers, the mean of the two ratings was calculated separately for grade and stage. The total OARSI score used in the statistical analyses was subsequently calculated by multiplying the resulting mean grade by the resulting mean stage. For the interobserver reliability analysis, a total OARSI score was also calculated separately for each pathologist by multiplying that observer’s grade and stage scores.

### 2.7. Statistical Analysis

An a priori sample size analysis was performed using G*Power version 3.1 (Heinrich-Heine-University, Düsseldorf, Germany). Based on an effect size of Cohen’s f = 0.59 derived from a previous experimental OA study evaluating intra-articular bevacizumab, an alpha error probability of 0.05, a statistical power of 80%, and four experimental groups, the minimum required total sample size was calculated as 36 animals using a fixed-effects one-way analysis of variance model [18]. Because no previous experimental OA study had compared all three anti-VEGF agents at the time of study design, the calculation was based on the only available experimental study investigating intra-articular bevacizumab in OA.

Statistical analyses were performed using IBM SPSS Statistics for Windows, version 26.0 (IBM Corp., Armonk, NY, USA). The primary statistical comparison was the overall comparison of total OARSI scores across the four experimental groups. Overall comparisons of OARSI grade and stage were considered secondary analyses. Pairwise comparisons were considered exploratory and were performed only when the corresponding overall Kruskal–Wallis test was statistically significant. OARSI grade, OARSI stage, and total OARSI score were treated as ordinal variables and are presented as median and interquartile range (IQR). Because the primary and secondary outcomes consisted of ordinal histopathological scores and the sample size was relatively small, non-parametric statistical methods were selected a priori. Comparisons among the four experimental groups were performed using the Kruskal–Wallis *H* test. When a significant overall difference was identified, pairwise comparisons were conducted using Dunn’s post hoc test with Holm adjustment for multiple comparisons. Effect sizes for the Kruskal–Wallis tests were calculated using epsilon-squared $\left(\varepsilon^{2}\;=\;\frac{H-k+1}{n-k}\right)$, where H represents the Kruskal–Wallis test statistic, *k* the number of groups, and *n* the total sample size. Interobserver agreement was assessed separately for OARSI grade, OARSI stage, and total OARSI score using a two-way random-effects, absolute-agreement, single-measures intraclass correlation coefficient [ICC(2,1)] with 95% confidence intervals. Because the final histopathological analyses were based on scores derived from the ratings of both pathologists, the corresponding average-measures coefficients [ICC(2,2)] were also calculated. All statistical tests were two-tailed, and a *p*-value < 0.05 was considered statistically significant.

## 3. Results

Three animals were lost during anesthesia and surgical induction of OA before allocation to the treatment groups. Consequently, 33 animals underwent random allocation and were included in the final analysis: bevacizumab (*n* = 8), ranibizumab (*n* = 8), aflibercept (*n* = 9), and control (*n* = 8). No additional animals were lost after treatment allocation.

Interobserver agreement assessed using ICC(2,1) was 0.766 (95% CI, 0.580–0.877) for OARSI grade, 0.684 (95% CI, 0.451–0.830) for OARSI stage, and 0.825 (95% CI, 0.674–0.910) for total OARSI score. The corresponding average-measures coefficients [ICC(2,2)] were 0.868 (95% CI, 0.734–0.934), 0.813 (95% CI, 0.622–0.907), and 0.904 (95% CI, 0.805–0.953), respectively.

Histopathological findings are summarized in Table 1 and Table 2. The primary analysis demonstrated a significant overall difference in total OARSI scores among the four groups (*H*(3) = 9.29, *p* = 0.026, ε^2^ = 0.217). Significant overall differences were also observed for OARSI grade (*H*(3) = 8.20, *p* = 0.042, ε^2^ = 0.179) and OARSI stage (*H*(3) = 9.93, *p* = 0.019, ε^2^ = 0.239) (Table 1).

Pairwise comparisons using Dunn’s test with Holm correction demonstrated significantly higher OARSI stage values in the aflibercept group than in the ranibizumab group (adjusted *p* = 0.014). Similarly, the aflibercept group exhibited significantly higher total OARSI scores than the ranibizumab group (adjusted *p* = 0.038). No other pairwise comparisons remained statistically significant after Holm adjustment (Table 2).

## 4. Discussion

The present study compared the histopathological effects of intra-articular bevacizumab, ranibizumab, and aflibercept in a surgically induced rat OA model using the OARSI histopathological grading system. Significant overall differences were observed among the experimental groups for OARSI grade, OARSI stage, and total OARSI score. Ranibizumab demonstrated numerically lower OARSI grade, stage, and total scores than aflibercept. Compared with the control group, ranibizumab demonstrated numerically lower OARSI grade and total OARSI scores, whereas both groups had the same median OARSI stage score. Bevacizumab demonstrated numerically lower OARSI grade and total scores than the control and aflibercept groups, whereas the median OARSI stage score was slightly higher than that of the control group. Pairwise analysis identified significantly lower OARSI stage and total OARSI scores in the ranibizumab group compared with the aflibercept group, while no statistically significant differences were observed between the treatment groups and the control group after adjustment for multiple comparisons. These findings indicate that the histopathological responses observed under the selected dosing conditions differed among the three agents; however, they do not demonstrate a protective effect of ranibizumab or bevacizumab because neither agent differed significantly from the saline control group.

When expressed on a molar basis for a representative 250 g rat, the administered doses corresponded to approximately 3.4 nmol of bevacizumab, 2.6 nmol of ranibizumab, and 7.0 nmol of aflibercept, based on their approximate molecular weights of 149, 48, and 115 kDa, respectively. Thus, aflibercept was administered at approximately 2.7 times the molar dose of ranibizumab. Consequently, the significant pairwise differences in OARSI stage and total OARSI score between the ranibizumab and aflibercept groups cannot be attributed solely to agent-specific biological effects, because they may also have been influenced by the unequal molar exposure.

The numerical reductions in OARSI grade and total score observed in the bevacizumab group are partially consistent with previous experimental studies supporting VEGF inhibition as a potential disease-modifying strategy for OA. Nagai et al. demonstrated that intra-articular bevacizumab significantly reduced cartilage degeneration, suppressed osteophyte formation, and improved histopathological findings in a rabbit OA model, suggesting that inhibition of VEGF-mediated angiogenesis may attenuate structural disease progression [14]. Similarly, Li et al. reported that intra-articular bevacizumab effectively attenuated articular cartilage degeneration and synovial hyperplasia in a rabbit model of knee OA. In addition, bevacizumab significantly reduced synovial VEGF and cartilage MMP-1 expression and demonstrated superior chondroprotective effects compared with sodium hyaluronate and triamcinolone acetonide, further supporting its anti-inflammatory and anti-catabolic potential [18]. More recently, Vadalà et al. reported that intra-articular bevacizumab significantly reduced OARSI histopathological scores in a dose-dependent manner while simultaneously increasing type II collagen and aggrecan expression and decreasing MMP-13 expression, indicating preservation of cartilage extracellular matrix and suppression of cartilage catabolism. The authors concluded that bevacizumab effectively arrested OA progression in their experimental rabbit model, with the highest dose producing the greatest histopathological improvement [22]. In a subsequent rat study, Çelik et al. evaluated intra-articular bevacizumab in a rat model of post-traumatic OA and reported significantly lower OARSI histopathological scores in bevacizumab-treated animals compared with untreated OA controls, with a dose-dependent reduction in cartilage degeneration. The authors concluded that VEGF inhibition attenuated structural cartilage damage and emphasized the need for larger experimental studies to validate these preliminary findings [23].

These experimental observations are further supported by the ex vivo study of Sotozawa et al., who demonstrated that bevacizumab directly suppressed cartilage catabolism in human osteoarthritic cartilage explants. Bevacizumab reduced the expression of several matrix metalloproteinases (MMP-2, MMP-3, MMP-9, and MMP-13), decreased cartilage degradation markers, and increased COL2A1 expression, suggesting both anti-catabolic and pro-anabolic effects on osteoarthritic cartilage [24]. Although the present study demonstrated numerically lower OARSI grade and total scores in the bevacizumab and ranibizumab groups, the median OARSI stage score in the ranibizumab group was identical to that of the control group. None of these differences was statistically significant compared with the control group after adjustment for multiple comparisons. Therefore, our findings provide only limited support for these previous experimental observations and should be interpreted cautiously.

The clinical relevance of the observed histopathological differences remains uncertain. Although OARSI scoring provides a standardized assessment of cartilage structural damage, differences in histological scores do not necessarily translate directly into differences in pain or joint function. Osteoarthritis pain and disability arise from interactions among multiple joint tissues, including the synovium, subchondral bone, menisci, ligaments, and infrapatellar fat pad, rather than from cartilage damage alone. Future experimental studies should therefore combine histopathological assessment with longitudinal measures of pain-related behavior, gait, weight-bearing asymmetry, locomotor activity, and joint function to determine whether structural differences are accompanied by clinically relevant functional benefits.

The present study was designed to compare histopathological outcomes and did not include molecular or immunohistochemical analyses. Therefore, it remains unclear whether the observed differences among the anti-VEGF agents were accompanied by differences in local VEGF suppression, matrix degradation, inflammation, or angiogenesis. Future mechanistic studies should incorporate assessments of VEGF expression and downstream signaling, cartilage-degrading enzymes such as matrix metalloproteinases, and endothelial markers or microvessel density as indices of angiogenesis. Combining these measurements with histopathological and functional outcomes would help determine whether structural differences reflect effective local VEGF inhibition and identify the biological pathways involved.

Increasing experimental evidence indicates that VEGF contributes to multiple pathological processes in OA, including angiogenesis, cartilage matrix degradation, chondrocyte hypertrophy, synovitis, and subchondral bone remodeling [4,6,7,10,25]. Consequently, VEGF inhibition has been proposed as a potential disease-modifying strategy for OA, although its therapeutic efficacy remains to be fully established [7,10].

Experimental studies evaluating ranibizumab in OA remain scarce. In the present study, the ranibizumab group exhibited numerically lower histopathological scores than the aflibercept group; however, no statistically significant difference was observed compared with the control group after adjustment for multiple comparisons. Therefore, these findings should be interpreted cautiously and require confirmation in future experimental studies.

The biological effects of VEGF inhibition are likely influenced by the complexity of VEGF signaling. Experimental studies have demonstrated that VEGFR1 and VEGFR2 contribute to different aspects of OA pathology, with VEGFR1 being more closely associated with pain signaling and VEGFR2 with cartilage degeneration. In addition, VEGF has been shown to stimulate matrix metalloproteinase production in osteoarthritic chondrocytes, supporting its role in cartilage matrix degradation [25,26,27,28]. These findings suggest that differences among anti-VEGF agents may depend not only on VEGF neutralization itself but also on receptor-specific downstream signaling pathways.

An intriguing finding of the present study was that aflibercept did not demonstrate superior histopathological outcomes despite its higher binding affinity for VEGF. Unlike bevacizumab and ranibizumab, aflibercept is a recombinant fusion protein that binds VEGF-A, VEGF-B, and placental growth factor (PlGF) with high affinity [11,12,29]. However, broader ligand binding does not necessarily translate into greater biological efficacy within the osteoarthritic joint. Experimental and pharmacokinetic studies suggest that tissue distribution, receptor occupancy, local drug exposure, and the complex joint microenvironment may influence the biological response to VEGF inhibition. In particular, Avery et al. demonstrated marked pharmacokinetic differences among anti-VEGF agents following intravitreal administration, with aflibercept exhibiting more prolonged systemic VEGF suppression than ranibizumab or bevacizumab [13]. Although these observations were obtained after ocular administration, they suggest that intrinsic pharmacokinetic differences may also contribute to variations in biological responses following intra-articular administration [6,11,12,30,31].

Because aflibercept did not differ significantly from the control group, the observed differences between ranibizumab and aflibercept should not be interpreted as evidence of a protective effect of ranibizumab alone. An alternative explanation is that aflibercept may have been associated with less favorable histopathological outcomes under the conditions of this experimental model. However, the present study was not designed to distinguish between these possibilities, and the limited sample size precludes definitive conclusions regarding potential harm.

Given the absence of previous experimental studies directly comparing bevacizumab, ranibizumab, and aflibercept in the same OA model, the present findings should be interpreted as preliminary evidence that anti-VEGF agents may not exert equivalent biological effects in osteoarthritic joints. Additional studies investigating intra-articular pharmacokinetics, tissue distribution, receptor activation, and downstream molecular responses will be required to clarify the mechanisms responsible for these differences. The local safety of intra-articular anti-VEGF administration also requires careful consideration. Although VEGF contributes to pathological angiogenesis and inflammation in OA, it also participates in physiological vascular homeostasis and tissue repair. Excessive or prolonged local VEGF inhibition could therefore theoretically affect synovial and subchondral vascular responses, cartilage homeostasis, or tissue healing. No predefined humane endpoint requiring euthanasia was reached during the present study; however, routine clinical monitoring alone is insufficient to establish local or systemic safety. Dedicated safety studies should include assessments of synovial inflammation and necrosis, chondrocyte viability, cartilage toxicity, subchondral bone and vascular changes, wound healing, systemic exposure, and potential off-target effects.

The present study has several important strengths. To our knowledge, this is the first experimental study directly comparing bevacizumab, ranibizumab, and aflibercept within the same surgically induced OA model under standardized experimental conditions. In addition, histopathological evaluation was performed independently by two blinded pathologists using the validated OARSI grading and staging system, providing a reliable and standardized assessment of cartilage degeneration. These methodological strengths enhance the internal validity of the present findings despite the exploratory nature of the study.

This study has several limitations. First, this was an experimental animal study, and although the anterior cruciate ligament transection model reproduces many pathological features of human OA, the findings cannot be directly extrapolated to clinical practice because of inherent biological and biomechanical differences between animal models and humans. Second, histopathological evaluation was performed at a single endpoint after treatment, precluding assessment of the temporal progression of cartilage degeneration and treatment response. Third, pain-related behavior, gait, weight-bearing asymmetry, locomotor activity, and joint function were not assessed; therefore, the clinical and functional relevance of the observed histopathological differences could not be determined. Biochemical, molecular, and immunohistochemical assessments of VEGF expression and downstream signaling, matrix metalloproteinases, inflammatory mediators, endothelial markers, and local microvessel density were not performed. Consequently, local target engagement, changes in cartilage matrix degradation, and the angiogenic or inflammatory mechanisms underlying the observed histopathological differences could not be determined. Furthermore, the three anti-VEGF agents were not administered at equimolar doses. For a representative 250 g rat, the molar dose of aflibercept was approximately 2.7 times that of ranibizumab. Therefore, the significant differences observed between these two groups may have been confounded by unequal molar exposure. Future studies incorporating equimolar treatment arms, multiple dose levels, and pharmacokinetic assessment are needed to distinguish agent-specific biological effects from dose-related effects. In addition, the Pritzker grade–stage system was used instead of the rodent-specific OARSI histopathology system. Although the Pritzker system enabled standardized assessment of cartilage lesion severity and extent, its use may limit direct comparability with studies applying the rodent-specific OARSI recommendations. Finally, three animals were lost during anesthesia and surgical induction of OA before treatment allocation, reducing the final sample size from 36 to 33. No post hoc power analysis was performed following these losses. The reduced sample size may have limited the statistical sensitivity of the study, particularly for detecting small between-group differences and pairwise differences after adjustment for multiple comparisons.

## 5. Conclusions

In conclusion, none of the anti-VEGF agents demonstrated a statistically significant histopathological improvement compared with the control group after adjustment for multiple comparisons. Although ranibizumab showed significantly lower OARSI stage and total scores than aflibercept, no significant differences were observed between any treatment group and the control group. Taken together, the present findings indicate that intra-articular VEGF inhibition did not produce a statistically significant histopathological improvement compared with saline under the dosing regimen and evaluation time point used in this study. Nevertheless, the observed differences between anti-VEGF agents suggest that these agents may not exert identical biological responses within the osteoarthritic joint. These observations should be considered exploratory and hypothesis-generating rather than confirmatory and require validation in larger experimental studies incorporating functional, molecular, and pharmacokinetic assessments.

## Figures and Tables

**Table 1:** Histopathological OARSI scores according to treatment groups.

Variable	Bevacizumab (*n* = 8)	Ranibizumab (*n* = 8)	Aflibercept (*n* = 9)	Control (*n* = 8)	*H* (df = 3)	*p*	ε^2^
OARSI Grade	2.0 (1.0–2.0)	2.0 (2.0–2.25)	3.0 (2.0–3.0)	3.0 (1.75–4.0)	8.20	0.042	0.179
OARSI Stage	2.5 (2.0–3.0)	2.0 (1.75–2.0)	3.0 (2.0–3.0)	2.0 (2.0–3.0)	9.93	0.019	0.239
Total OARSI Score	4.0 (3.0–4.5)	4.0 (2.75–4.0)	9.0 (4.0–9.0)	7.0 (2.75–8.25)	9.29	0.026	0.217

Values are presented as median (interquartile range). Overall group comparisons were performed using the Kruskal–Wallis test (df = 3). Epsilon-squared (ε^2^) is reported as the effect-size estimate.

**Table 2:** Pairwise comparisons of OARSI scores using Dunn’s test with Holm adjustment.

Comparison	Grade	Stage	Total Score
Bevacizumab vs. Ranibizumab	0.714	0.128	0.762
Bevacizumab vs. Aflibercept	0.061	0.919	0.077
Bevacizumab vs. Control	0.110	0.919	0.666
Ranibizumab vs. Aflibercept	0.699	**0.014**	**0.038**
Ranibizumab vs. Control	0.714	0.502	0.666
Aflibercept vs. Control	0.831	0.502	0.666

Adjusted *p* values are based on Dunn’s post hoc test with Holm correction for multiple comparisons. Statistically significant adjusted *p* values (*p* < 0.05) are shown in bold.

## Data Availability

The data presented in this study are available from the corresponding author upon reasonable request.

## Abbreviations

The following abbreviations are used in this manuscript:

OA	Osteoarthritis
VEGF	Vascular Endothelial Growth Factor
VEGFR	Vascular Endothelial Growth Factor Receptor
PlGF	Placental Growth Factor
ACLT	Anterior Cruciate Ligament Transection
OARSI	Osteoarthritis Research Society International
IQR	Interquartile Range
H&E	Hematoxylin and Eosin
MMP	Matrix Metalloproteinase
IgG1	Immunoglobulin G1
